# Nucleostemin mRNA is expressed in both normal and malignant renal tissues

**DOI:** 10.1038/sj.bjc.6603145

**Published:** 2006-05-02

**Authors:** Y Fan, Z Liu, S Zhao, F Lou, S Nilsson, P Ekman, D Xu, X Fang

**Affiliations:** 1 Department of Surgery, Division of Urology, Division of Hematology, Karolinska University Hospital, SE-171 76 Stockholm, Sweden; 2 Department of Urology, Qilu Hospital, Shandoing University, Jinan, 250012, PR China; 3 Department of Oncology-Pathology, Division of Hematology, Karolinska University Hospital, SE-171 76 Stockholm, Sweden; 4 Aging and Health Center, Nursing School, Shandong University, Jinan, 250012, PR China; 5 Institute of Urology, Shandong University, Jinan, 250012, PR China; 6 Department of Medicine, Division of Hematology, Karolinska University Hospital, SE-171 76 Stockholm, Sweden

**Keywords:** nucleostemin, RCC, renal tissues, c-MYC

## Abstract

Nucleostemin (NS), a p53-binding protein, has been shown essential for stem and cancer cell proliferation and implicated in oncogenesis. To explore potential contributions of NS to the development of clear cell renal cell carcinomas (ccRCCs), we determined NS expression in ccRCC cell lines, and in paired normal and malignant renal tissues from 31 patients with ccRCC. Nucleostemin mRNA and/or protein expression was observed in all four cell lines and 27 of 31 (87%) tumour specimens. Surprisingly, 16 of 31 (52%) adjacent normal renal samples also expressed NS mRNA and its levels in four of them were comparable with those in paired tumour tissues. Three of the patients had detectable NS mRNA in their normal renal tissues whereas lacked its expression in the matched tumours. Compared to the oncogene c-MYC expression in these same samples, NS expression showed a much less specificity for ccRCC. We further demonstrated that NS mRNA expression was closely associated with cellular proliferation in normal fibroblasts or T lymphocytes and renal cell carcinoma cell lines. Collectively, NS expression widely occurs in normal and malignant renal tissues, and is likely a proliferation marker rather than a unique regulator of cell proliferation and survival in stem and cancer cells.

Renal cell carcinoma (RCC), the most common malignant tumour of the adult kidney in both developed and developing countries, accounts for 2–3% of all human malignancies with close to 100 000 yearly deaths worldwide (Whang and Godley, 2003; Lam *et al*, 2005; Patel *et al*, 2006). As a pathologically and genetically heterogeneous disease, RCC falls into at least four subtypes, including clear-cell, a predominant type (about 80%) and others (papillary, chromophobe, and collecting duct carcinomas). In clear cell renal cell carcinoma (ccRCC), inactivation of the von Hippel–Lindau disease tumour suppressor (*VHL* gene) by different mechanisms is widespread and believed to be an early or a first event during the oncogenic process (Foster *et al*, 1994; Gnarra *et al*, 1994; Brauch *et al*, 2000; Linehan *et al*, 2004; Maher, 2004). However, molecular mechanisms underlying the full development of ccRCC are largely unknown. Clinically, approximately 30% of patients with ccRCC present with widely disseminated diseases, and even higher percentages of patients treated with complete surgical resection undergo metastatic progression within a short period (Glaspy, 2002; Pantuck *et al*, 2003; Whang and Godley, 2003; Lam *et al*, 2005; Patel *et al*, 2006). Moreover, recurrent ccRCC responds poorly to the current biotherapy and chemotherapy (Donskov *et al*, 2006; Patel *et al*, 2006). Although a number of molecular and clinical parameters have been shown as prognostic markers for ccRCC (Campbell *et al*, 2003; Elmore *et al*, 2003; Kallio *et al*, 2003; Schips *et al*, 2003; Thomas *et al*, 2003; Kallio *et al*, 2004; Zigeuner *et al*, 2004; Dudderidge *et al*, 2005), the clinical outcome of patients with ccRCC shows a broad variation. Therefore, better defining the pathogenesis of ccRCC, looking for reliable early diagnostic markers, and exploring novel intervention targets for ccRCC are urgently demanding tasks.

One of the most important hallmarks for cancers is their deregulated cellular proliferation. The aberrant activation of the oncogene c-MYC, observed in many different types of human cancers, has been implicated as a driving force for abnormal cell growth (Nasi *et al*, 2001; Hermeking, 2003). More recently, a p53-binding protein, named nucleostemin (NS) abundantly expressed in stem and cancer cells but silenced in differentiated cells, has been shown to be essential for stem and cancer cell proliferation and survival (Tsai and McKay, 2002). It is currently unknown whether this newly identified molecule is involved in ccRCC pathogenesis. In the present study, we investigated NS mRNA expression in both normal and malignant renal tissues, and examined potential relationships between its expression and clinico-pathological characteristics in ccRCC. Additionally, because NS shares certain similarities with the oncogene c-MYC in terms of expression profile and functional properties (Nasi *et al*, 2001; Tsai and McKay, 2002), we further compared the NS expression profile with the *c-MYC* activation in ccRCC.

## PATIENTS AND METHODS

### Cell lines and cell culture

Four ccRCC cell lines, A498, KRC/Y, CAKI-2, and TK10, were maintained at 37°C in Dulbecco's modified Eagle's medium (Life Technologies, Paisley, UK) supplemented with 10% fetal calf serum (FCS), 2 mM L-glutamine, 100 U ml^−1^ penicillin and streptomycin, under 5% CO_2_/95% air. Cells at logarithmically growing phases were harvested for reverse transcription–polymerase chain reaction (RT–PCR) analyses of NS and c-MYC mRNA expression. To determine the relationship between NS expression and cellular proliferation, the cells were incubated in 0.2% FCS-containing medium for 72 h to induce quiescence and then re-fed with 20% FCS for 24 h. Normal T lymphocytes were isolated from the buffy-coaded blood derived from normal individuals and treated with 15 *μ*g/ml ConA to stimulate their proliferation. Normal dermal fibroblasts were kindly provided by Dr Z. Yan (Karolinska Institute, Sweden).

### Patients and tissue specimens

The ccRCC tumour samples and their adjacent normal renal tissues were obtained from 31 patients with primary ccRCC. The study was approved by the local ethics committee, and all the patients consented to the use of their renal tissues for the present investigation. The patients were treated with radical nephrectomy at Qilu Hospital, Shandong University, between 2002 and 2003. After surgery, the tumour specimens and adjacent normal renal tissues were stored at −80°C until use. For each ccRCC, tumour size (diameter), Robson stage, and regional and distant metastases at nephrectomy were evaluated by both pathologists and urologists. Patient and disease information is listed in Table 1.

### RNA extraction, reverse transcription, and polymerase chain reaction

Total cellular RNA was extracted using the ULTRASPEC™ -II RNA kit (Biotecx Lab., Houston, TX, USA). cDNA was synthesised using random primers (N6) (Pharmacia, Uppsala, Sweden) and M-MLV reverse transcriptase. The polymerase chain reaction (PCR) primer sequences specific for NS mRNA (Accession number: AY825265) were: 5′-GAA ACA GAG GCT TGA AGA ACT AA (forward) and 5′-GGA GGC TTC GAT CAC CTT TTT A (reverse). Polymerase chain reaction was performed with the use of 32 amplification cycles (95°C for 15″, 64°C for 30″, and 72°C for 30″) and the PCR product was 223 bp in length. The RT–PCR for the oncogene c-MYC mRNA (Accession number: HSMYC1) was performed as described (Garrett *et al*, 2002), and the primer sequences were: forward: TAC CCT CTC AAC GAC AGC AGC TCG CCC AAG TCC T and reverse: TCT TGA CAT TCT CCT CGG TGT CCG AGG ACC T, which led to the amplification of a 479 bp long DNA fragment. A total of 30 PCR cycles at 95°C for 15″, 60°C for 45″, and 72°C for 60″ was carried out. *β*-Actin expression was used as an internal control and amplified with its specific primers using 25 cycles (Fan *et al*, 2005). Polymerase chain reaction products were resolved in 2% agarose gels, stained with ethidium bromide, and visualized in ultraviolet light. The PCR conditions were optimised to make sure a linear amplification of each target, and PCR results for NS and c-MYC were recorded as negative (−), moderate positive (+), and strongly positive (++) based on their signal intensities and *β*-actin reference.

### Western blot

Western blot was performed as described (Xu *et al*, 2000). Briefly, total proteins were extracted with sodium dodecyl sulphate lysis buffer, and 50 *μ*g of the proteins were resolved by sodium dodecyl sulphate–polyacrylamide gel electrophoresis and transferred to an intracellulos membrane. The membranes were probed with the specific primary antibody against NS (Cat# 14-86-42-80; eBioscience, San Diego, CA, USA) followed by secondary anti-rabbit horseradish peroxidase-conjugated IgG and developed with the enhanced chemiluminescent method (ECL, Amersham, UK).

### Immunohistochemistry

Immunohistochemical staining was performed as described earlier (Zhang *et al*, 2000). Following deparaffinization, antigen unmasking, and inactivation of endogenous peroxidase, the primary antibody against NS (eBioscience, San Diego, CA, USA) was 1:10 diluted, added onto slides, and incubated at 4°C overnight. An antibody binding was visualised by using the secondary antibodies conjugated with horseradish peroxidase and an AEC kit according to the manufacturers' protocols (Dako Corporation, Denmark). The slides were finally counterstained with haematoxylin. Coverslips were mounted onto glass slides and examined with a light microscope equipped with a CCD camera (Nikon, Eclipse E800, Japan).

## RESULTS

### NS mRNA and protein expression in ccRCC cell lines

**Figure 1:**
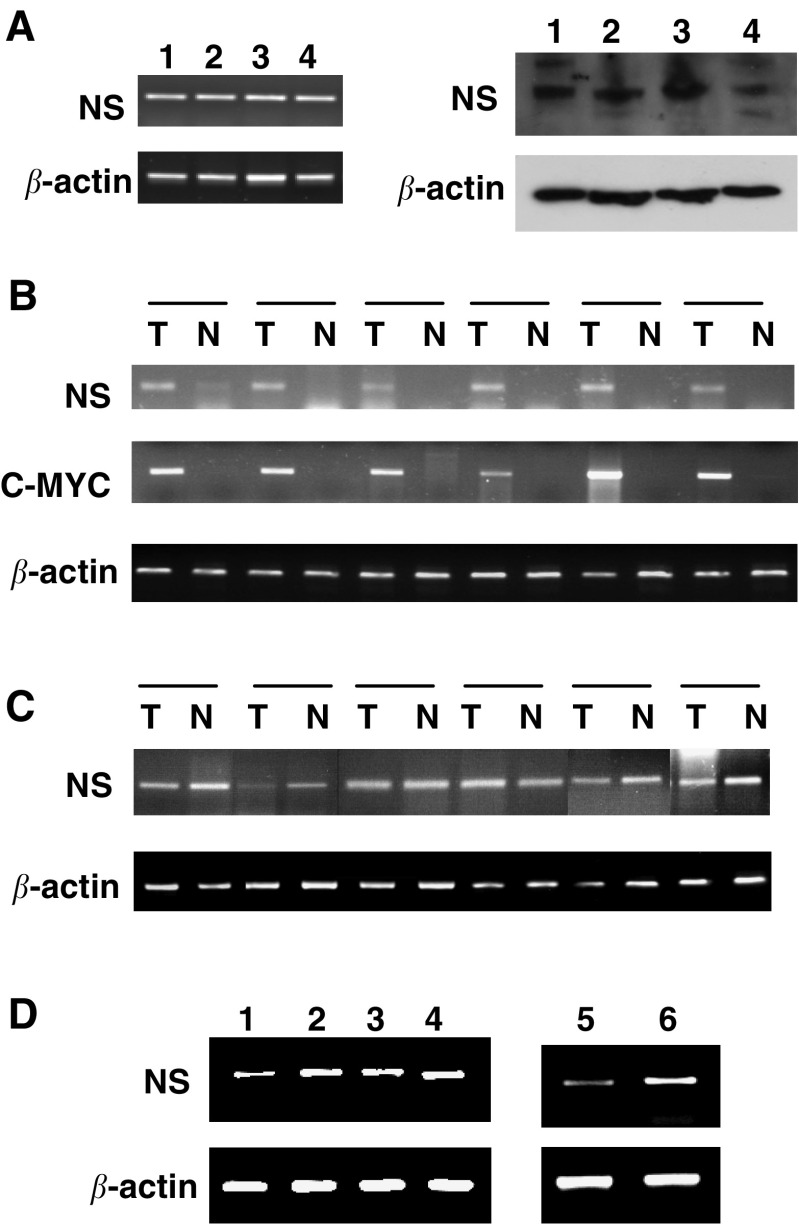
Nucleostemin expression in normal and malignant renal tissues and other types of normal human cells. (**A**) Nucleostemin mRNA (left panel) and protein (right panel) expression in ccRCC cell lines. Lane 1: A498 cells; 2: TK10; 3: KRY/C, and 4: Caki2. (**B**) and (**C**) NS and c-MYC mRNA expression in ccRCC samples and their adjacent normal renal tissues. (**D**) Nucleostemin mRNA expression in normal human fibroblasts, T lymphocytes, and A498 cells and relationship with cell proliferation statuses. Lanes 1 and 2: normal fibroblasts at senescence and at early passage, respectively. Lanes 3 and 4: normal resting and activated T lymphocytes, respectively. Lane 5: ccRCC A498 cells with serum-starvation for 72 h. Lane 6: A498 cells undergoing 72 h serum-starvation were re-fed with 20% serum for 24 h.

We first examined NS mRNA and protein in cultured ccRCC cell lines. For this purpose, A498, KRC/Y, CAKI-2, and TK10 cells were used and all the four cell lines contained NS mRNA, as determined by using RT–PCR (Figure 1A, left panel). The result demonstrated that ccRCC tumour-derived cells, like other types of human cell lines reported previously, exhibited a transcriptional activation of the *NS* gene. Consistent with its mRNA expression, NS protein was readily detectable in all these cell lines with the use of an immunoblotting assay (Figure 1A, right panel).

### NS mRNA and protein expression in primary ccRCC specimens

**Figure 2:**
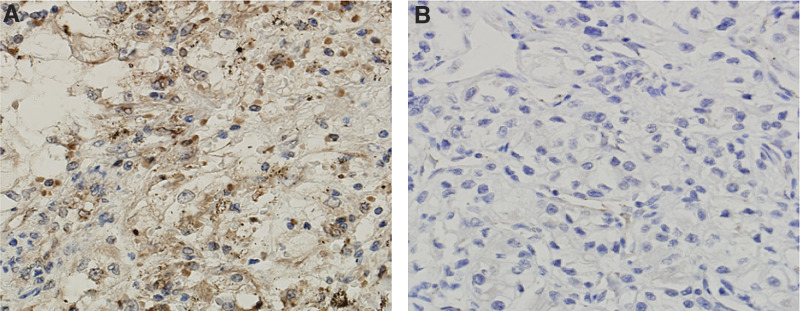
Immunohistochemical analyses for NS protein expression in tumour specimens derived from patients with ccRCC. Representative positive (**A**) and negative (**B**) stainings are shown.

We next carried out NS mRNA analyses in primary ccRCC samples. A total of 31 tumours obtained from 31 patients with ccRCC were examined and NS mRNA detected in 27 of them (86%, Table 1, Figure 1B and C). Compared to the cell lines, those primary tumours had largely similar levels of NS mRNA. Moreover, we further performed immunohistochemical analyses on 10 available tumour specimens to examine NS protein expression. Nine of 10 examined patient tumours exhibited a positive staining for NS in more than 20% cells and in all the positive specimens, NS was observed in both cytoplasmic and nuclear compartments of tumour cells (Figure 2). This distribution profile of NS was very similar to that found in gastric cancers (Yang *et al*, 2005). The staining intensity of the NS protein was in general consistent with its mRNA expression in the examined tumour specimens.

We then compared NS expression with clinico-pathological parameters, and found no significant associations between the presence of NS mRNA and patients' age, gender, tumour sizes, disease stages, and metastases.

### Detectable NS mRNA expression in more than half of adjacent normal renal tissues

We then wanted to ask whether transcriptional activation of the *NS* gene also occurred in normal renal cells. The adjacent normal renal samples from these 31 patients were analysed for NS mRNA expression, and unexpectedly, 16 of them (51%) expressed detectable NS mRNA (Table 1 and Figure 1C). Of these NS-expressing normal samples, the levels of NS mRNA in nine cases were lower than in their paired tumours and in four patients were comparable with their tumour counterparts. Intriguingly, it was observed in three patients that NS mRNA was only present in their normal renal tissues whereas absent in their tumour specimens (Table 1, patients 4, 6, and 29). Taken together, NS mRNA is expressed in normal human renal tissues with a high frequency.

### A close relationship between NS expression and cell proliferation in normal human cells and ccRCC cells

To determine whether other kinds of normal cells express NS, we analysed NS mRNA in normal human T lymphocytes and dermal fibroblasts. As shown in Figure 1D, NS mRNA was readily detectable in both cell types. We further observed that T lymphocytes activated by ConA expressed higher NS mRNA than did the resting cells, and that senescent fibroblasts had lower abundances of NS mRNA compared to the cells at early passages.

The above result suggests that NS may be a universal proliferation marker rather than a specific regulator for proliferation of undifferentiated stem and cancer cells. To test this hypothesis, we examined the kinetic changes in ccRCC cell lines at quiescent and actively proliferating phases. We first induced quiescence of A498 cells by maintaining them in 0.2% of FCS for 72 h and then stimulated their proliferation by transferring them into 20% FCS-containing medium. The RT–PCR analyses clearly demonstrated significant downregulation of NS expression in G0 cells, but increased NS mRNA abundance following re-entry into cell cycle (Figure 1D).

### The oncogene c-MYC expression in normal and cancerous renal tissues

We finally analysed c-MYC mRNA expression in this same set of patients' specimens. c-MYC mRNA was found in all 29 examined tumour samples. Twenty-one of 29 adjacent normal renal tissues lacked detectable c-MYC mRNA (Table 1 and Figure 1B). Although eight of those normal samples expressed c-MYC mRNA, the expression level was much lower compared to that in their paired tumour tissues. We found no associations between NS and c-MYC expression in both normal and malignant renal tissues, indicating independent transcriptional regulation of two genes.

## DISCUSSION

Tsai and McKay (2002) have recently identified NS, a novel p53-binding protein that is abundantly present in stem cells and stem cell-enriched tissues. Interestingly, both NS mRNA and protein are absent in adult differentiated cells/tissues, although highly expressed in different human cancer cell lines (Tsai and McKay, 2002). Depletion of NS inhibits cell proliferation in both stem and cancer cells, indicating its functional importance in maintaining cell cycle progression of those cells. Prompted by these interesting findings, we set out to investigate whether NS has roles in RCC development, and if so, whether this molecule can serve as a diagnostic marker or therapeutic target for RCC.

In the present study, we observed NS expression in all four examined ccRCC cell lines and in 27 of 31 primary tumours, suggestive of its widespread in primary ccRCC, consistent with that seen in human cancer cell lines. Surprisingly, more than the half of adjacent normal renal specimens also contained NS mRNA, and the expression level in four of them was comparable with their paired tumour tissues. In three patients, NS mRNA was present in their normal renal tissues but absent in their tumours. Moreover, we have similarly observed that both normal and cancerous prostate tissues expressed rather high levels of NS mRNA (X Fang, P Ekman, and D Xu, unpublished data). These unexpected findings challenge the early concept of NS being a specific player for stem and cancer cell proliferation. However, one may argue that (1) tumour cell contamination in adjacent normal renal tissues may contribute to detectable NS mRNA expression, as seen here. However, these same specimens have previously been used for analyses of telomerase activity and full-length of telomerase reverse transcriptase mRNA expression, highly specific markers for transformed cells, and both markers were exclusively absent in all the studied adjacent normal tissues (Fan *et al*, 2005), which suggests the lack of contaminated tumour cells in the normal samples used in this study. (2) The stem cells present in kidneys and prostates may be a source of NS mRNA. However, stem cells in somatic tissues kidneys and prostates are in a rather low number, and are unlikely a major contributor to the observed NS expression in these organs. Our further identification of NS mRNA expression in purified T lymphocytes and fibroblasts strongly indicate that many types of differentiated human cells, if not all, do express NS transcripts. Taken together, we conclude that NS expression is not a unique feature for ccRCC and prostate cancers, neither for stem cells.

According to our preliminary data, NS expression is proliferation-regulated and induced in actively cycling cells such as presenescent human fibroblasts and activated T lymphocytes. On the other hand, *in vitro* cultured RCC cells undergo downregulation of NS expression in response to serum starvation. Regardless being stem cells, cancer cells, or normal differentiated cells, they may all acquire NS expression when in actively proliferating status. Therefore, NS is more like a proliferation marker rather than a molecule only involved in the cell cycle control of stem and cancer cells. This proposal provides a reasonable explanation for what have been seen in normal renal and prostate tissues: detectable NS transcript in them is virtually expressed by proliferative epithelial or other types of cells; and NS-expressing stem cells are negligible owing to their very low numbers.

In normal stem cells and U2OS cells, NS protein was exclusively localised in nucleus (Tsai and McKay, 2002). However, the primary ccRCC tumour cells examined here exhibited both cytoplasmic and nuclear localisations of NS, although a nuclear compartment with a stronger signal. This distribution pattern of NS has recently been observed in gastric cancers, too (Yang *et al*, 2005). It is currently unclear how and why such a cytoplasmic localization occurs, which calls for further investigations.

The oncogene c-MYC plays a key role in regulating cell proliferation and its aberrant activation contributes to the pathogenesis of many human malignancies including RCC (Nasi *et al*, 2001). From expression and functional points of view, c-MYC and NS share certain similarities: (1) both are abundantly present in stem cells and downregulated in differentiated cells and (2) their expression levels should be within a limited window, and both depletion and overexpression lead to cell growth arrest and/or apoptosis. Thus, we determined NS and c-MYC mRNA expression in parallels. c-MYC mRNA was expressed in all 29 tumours examined, whereas lower or comparable levels of c-MYC expression was only observed in 8/29 normal renal samples. Compared to NS, c-MYC is a more specific marker for ccRCC.

The study presented herein demonstrates that NS is widely expressed in both ccRCC and adjacent normal renal tissues, in sharp contrast with the recent report that NS is present in stem and cancer cells whereas absent in differentiated adult tissues (Tsai and McKay, 2002). Although the levels of NS expression are higher in most tumours than their paired normal renal tissues, there was no significant correlation between NS expression and patients' clinico-pathological characteristics. Compared to the c-MYC oncogene, NS expression shows less specificity for ccRCC, and is unlikely to serve as a diagnostic marker and therapeutic target for RCC. Our findings call for re-evaluation of the NS expression profile in various normal human tissues, and more rigorous delineation of its oncogenic role in human cell transformation.

## Figures and Tables

**Table 1:** Patients' characteristics, nucleostemin, and c-MYC mRNA expression in ccRCCs and adjacent normal renal tissues

**Clinical/pathological information**				**Tumours**		**Normal tissues**	
**Patient**	**Age/gender**	**Sizes (cm)**	**Stages**	**NS^a^**	**c-MYC^a^**	**NS**	**c-MYC**
1	56/M	5.0	I	+	++	−	+
2	54/F	4.5	I	++	+	+	+
3	56/M	5.5	I	++	++	−	−
4	52/M	8.0	I	−	+	+	+
5	47/F	7.5	I	+	++	−	−
6	54/F	6.0	I	−	ND^2^	+	ND
7	54/F	10.0	I	++	+	−	−
8	64/M	8.0	I	−	+	−	−
9	68/F	4.5	I	++	++	−	−
10	57/M	6.5	I	+	++	−	−
11	76/M	5.0	I	+	+	+	−
12	44/M	2.5	I	++	++	−	−
13	62/M	3.0	I	+	+	−	−
14	48/F	3.0	I	++	++	+	−
15	74/M	3.0	I	++	++	+	+
16	57/F	4.0	I	++	++	+	−
17	51/M	3.0	I	+	+	+	+
18	68/M	6.0	I	++	+	−	−
19	79/F	3.0	I	++	++	+	−
20	36/M	6.5	I	++	+	−	−
21	48/F	8.0	I	++	++	+	−
22	43/M	4.5	I	+	++	+	−
23	63/M	4.5	I	+	+	−	−
24	39/F	5.0	II	+	+	+	+
25	48/M	8.0	II	++	++	+	−
26	68/M	10.5	II^M^^b^	++	ND	−	ND
27	69/M	8.0	II^M^	++	++	−	−
28	64/M	8.0	III^M^	++	++	+	+
29	54/M	12.5	IV^M^	−	+	+	−
30	28/F	15.5	IV^M^	++	++	+	+
31	64/M	7.0	IV^M^	+	+	−	−

ccRCCcs=clear cell renal cell carcinomas; ND=not done; NS=nucleostemin; PCR=polymerase chain reaction.

a PCR results for NS and c-MYC were recorded as negative (−), moderate positive (+), and strongly positive (++) based on their signal intensities.

b M: With metastasis.
